# Heat shock proteins 70/90 and associations with immunosuppression along with sepsis: preliminary data

**DOI:** 10.1186/cc14122

**Published:** 2015-03-16

**Authors:** P Papadopoulos, A Pistiki, T Christodoulopoulou, M Theodorakopoulou, V Tsagkari, A Armaganidis, S Tsiodras, I Dimopoulou, G Briassoulis, G Briassoulis

**Affiliations:** 1 University Hospital of Athens, Greece; 2 University Hospital ATTIKON, Athens, Greece; 3 University Hospital, University of Crete, Heraklion, Greece

## Introduction

CD14/HLADR is an index of immune suppression. Heat shock proteins (hsp) regulate cell response to oxidative stress. We evaluated the relationship of CD14/HLADR and hsp70/90 in patients with SIRS and severe sepsis versus healthy volunteers.

## Methods

We evaluated 31 patients with SIRS or severe sepsis against a group of sex-matched healthy volunteers. Demographic data were obtained for all patients. APACHE score was calculated upon admission. Blood samples were collected upon diagnosis of SIRS or severe sepsis. To evaluate the %HLA-DR expression on monocytes, the fresh whole blood was stained with anti-CD14-FITC, anti-HLA-DR-PE and CD45PC5 while staining with anti-CD33-PE, anti-CD45-PC7, anti-hsp70-FITC and anti-hsp90-PE allowed evaluation of the MFIexpression of hsps on CD33^+ ^monocytes. Cells were then analyzed using flow cytometry. ANOVA with *post hoc *tests was used to compare CD14/HLADR cell counts and hsp70 and hsp90 levels among the three groups.

## Results

Nineteen controls, six SIRS patients and 25 severe sepsis patients were studied. The percent expression of HLADR on CD14^+ ^monocytes was significantly different between the three groups showing progressive decrease from controls (mean 90.5 ± 3.8%) to SIRS (mean 61.2 ± 5.9%) to severe sepsis (mean 39.2 ± 5.5%) patients (controls vs. severe sepsis, *P *< 0.001; controls vs. SIRS, *P *= 0.006; SIRS vs. severe sepsis, *P *= 0.03). hsp70 and hsp90 MFIwere significantly different between controls (mean 49.5 ± 4.9 and 33.5 ± 3.4 respectively), SIRS (mean 69.9 ± 16.5 and 46.5 ± 5.7 respectively) and severe sepsis patients (mean 33.3 ± 4.5 and 21.7 ± 2.7 respectively) (*P *< 0.05 for all comparisons). Notably, the hsp level rose from controls to SIRS and fell from SIRS to severe sepsis patients. APACHE score increased significantly (*P *= 0.023) in septic patients compared with SIRS.

## Conclusion

There were a significant difference in CD14/HLADR, a marker of immune paralysis, between controls and patients with SIRS or severe sepsis. hsp70 and hsp90 showed an initial stimulation followed by exhaustion as sepsis progressed.

## Acknowledgements

This research was co-financed by the European Union (European Social Fund) and Greek national funds through the Operational Program 'Education and Lifelong Learning' of the National Strategic Reference Framework Research Funding Program: THALES. This abstract is part of the study 'Heat Shock Proteins and Glutamine Alterations Related to Hormonal, Immunological, Inflammatory and Molecular Response to Sepsis: A Combined Clinical and Experimental Study'.

